# Bioactivity-guided isolation and identification of anti-adipogenic compounds from *Sanguisorba officinalis*

**DOI:** 10.1080/13880209.2017.1357736

**Published:** 2017-08-23

**Authors:** Sun Hyuk Im, Zhiqiang Wang, Soon Sung Lim, Ok-Hwan Lee, Il-Jun Kang

**Affiliations:** a Department of Food Science and Nutrition, Hallym University, Chuncheon, Republic of Korea;; b Department of Food Science and Biotechnology, Kangwon National University, Chuncheon, Republic of Korea

**Keywords:** Anti-obesity, ellagic acid, isorhamnetin-3-*O*-d-glucuronide

## Abstract

**Context:***Sanguisorba officinalis* Linne (Rosaceae) is a medicinal plant used traditionally for the treatment of inflammatory and metabolic diseases in Korea, China, and Japan. In our previous study, a 50% ethanol extract inhibited fat accumulation in 3T3-L1 adipocytes.

**Objective:** This study investigates bioassay-guided fractionation, isolation, and identification of anti-adipogenic bioactive compounds in *S. officinalis*.

**Materials and methods:** The bioassay-guided fractionation was conducted using effective differentiation of 3T3-L1 cells into adipocytes (with 50 μg/mL test material for 8 days) to isolate the inhibitory compounds from ethyl acetate fraction of *S. officinalis* 50% ethanol extract. The cytotoxicity of each fraction and isolated compound was tested using MTT assay (with 25–300 μg/mL test material). Structures of the isolated active compounds were elucidated using ^1^H NMR, ^13 ^C NMR, HSQC, HMBC, FT-IR, and MS.

**Results:** An active ethyl acetate fraction obtained with solvent partition of the extract inhibited lipid accumulation (44.84%) on 3T3-L1 cells without cytotoxicity (102.3%) at the concentration of 50 μg/mL. The ethyl acetate fraction was determined to be mainly composed by isorhamnetin-3-*O*-d-glucuronide (1) and ellagic acid (2). Pure isorhamnetin-3-*O*-d-glucuronide (IC_30_ is 18.43 μM) and ellagic acid (IC_30_ is 19.32 μM) showed lipid accumulation inhibition on 3T3-L1 cells without cytotoxicity (117.5% and 104.3%) at the concentration of 20 μM, respectively.

**Discussion and conclusions:** These results suggested that *S. officinalis* is a potential natural ingredient for the prevention of obesity, which may due to bioactive compounds such as isorhamnetin-3-*O*-d-glucuronide and ellagic acid.

## Introduction

Obesity, as a multifactorial and chronic disease, is a serious public health problem worldwide that increases the risk of type 2 diabetes, hypertension, cardiovascular diseases, cancers, and nonalcoholic fatty liver disease (Visscher and Seidell 2001). The World Health Organization (WHO) indicated that 600 million adults are obese, and 41 million children are overweight or obese, as of 2014 (WHO 2017). Obesity is defined as a medical condition with an abnormal accumulation of body fat, and it is associated with excessive growth and an expansion of adipose tissue. The cause of obesity is very complex, affected by genetics, as well as some environmental factors such as lifestyle, the cost of energy-dense foods, and levels of physical activity, but it can simply be based on an imbalance between energy intake and energy expenditure (Williamson et al. 1993; Kopelman and Grace 2004). It may be characterized by increased adipose tissue mass that results from the differentiation of preadipocytes into an excess number and size of adipocytes via the process of adipogenesis (Spiegelman and Flier 2001). Recent reports have recognized the mechanisms of proposed anti-obesity, including inhibited adipogenesis, decreased lipogenesis, and increased lipolysis (Hsu and Yen 2006; Rayalam et al. 2008). Regulating adipogenesis, therefore, provides a promising therapeutic approach for preventing obesity.

Natural plants and foods have been used globally as a complementary and alternative medicine (Recio et al. 2012). Several plants have been reported to help prevent obesity through regulating adipogenesis, indicating that dietary bioresources, such as medicinal foods and functional ingredients, may potentially have protective roles against adipocyte differentiation and fat accumulation in adipose tissue (Hsu and Yen 2006). Our research group recently reported the anti-obesity effect of *Solidago virgaurea* through regulating adipogenesis and have identified protocatechuic acid, chlorogenic acid, and kaempferol-3-*O*-rutinoside as the major active components from *S. virgaurea* using bioactivity-guided isolation approaches (Jang et al. 2016; Wang et al. 2017). These results encouraged us to seek additional functional ingredients from natural origins.

*Sanguisorba officinalis* Linne (Rosaceae) (SOL) is a perennial plant that grows wild on hills and in fields (Lee et al. 2004). The height of the plant ranges from 0.3 to 0.15 m, the rhizome grows lengthwise and sideward, the stem grows straight, the leaves are a serrate oval shape, and it has the scent of cucumber (Lee, Oh, et al. 2010). The flowers are dark red, they bloom from July to September, and the quadrangle fruits ripen in October (Kim et al. 2000). Its young leaves are edible and the roots are used as a haemostatic agent (Park et al. 2004). SOL has various anti-inflammatory, anti-allergic, and anticancer effects as shown in previous studies (Shin et al. 2002, 2012; Lee, Lee, et al. 2010). We previously reported (Jung et al. 2016) that a 50% ethanol (EtOH) extract from SOL considerably inhibited the triacylglycerol accumulation through the down-regulation of peroxisome proliferator-activated receptor-γ (PPAR-γ), CCAAT/enhancer binding protein-α (C/EBPα), and sterol regulatory element-binding protein (SREBP-1c) in 3T3-L1 cells. Additionally, the 50% EtOH extract reduced body weight and adipose tissue weight, and improved serum lipid profiles through the down-regulation of PPAR-γ, C/EBPα, fatty acid binding protein-4 (FABP4), and acetyl-CoA carboxylase (ACC), and up-regulation of adiponectin and carnitine palmitoyltransferase 1 (CPT-1) in high-fat diet obese C57BL/6 J mice. However, the related chemical constituents of the SOL 50% EtOH extract responsible for the anti-obesity activity remain unclear. Therefore, in the present study, we attempted to isolate and identify the major anti-adipogenic constituents from SOL by bioassay-guided fractionation. Two compounds were thus obtained, and their anti-adipogenic effect was evaluated in 3T3-L1 cells.

## Materials and methods

### Materials

The SOL, harvested in July 2015, was supplied by Gidam Ltd. (Inje, Korea). 3T3-L1 preadipocytes were obtained from the American Type Culture Collection (ATCC, CL-173, Manassas, VA). Dulbecco’s modified Eagle’s medium (DMEM), bovine calf serum (BCS), foetal bovine serum (FBS), penicillin–streptomycin (P/S), phosphate-buffered saline (PBS), and trypsin–EDTA were purchased from Gibco (Gaithersburg, MD). Dexamethasone (DEX), dimethyl sulphoxide (DMSO), 3-isobutyl-1-methylxanthine (IBMX), insulin, and Oil Red O were purchased from Sigma (St. Louis, MO). Unless otherwise specified, all other chemicals were purchased from Sigma.

### Extraction, fractionation, and isolation of bioactive compounds from SOL

Fresh cleaned SOL was dried at 40 °C for 6 h and then ground to a powder. SOL powder (1 kg) was mixed with 50% EtOH (10 L) and refluxed three times (2 h each time) at 80 °C. The extracts were then filtered using Whatman filter paper (No. 2). The filtrate was concentrated *in vacuo* and completely dried with a freeze drier (ilShin Lab. Co., Ltd., Seoul, Korea). The freeze-dried EtOH extract (279 g) was dissolved in water (900 mL) and fractionated with 300 mL of *n*-hexane (*n*-Hex), methylene chloride (MC), ethyl acetate (EA), *n*-butanol (*n*-BuOH), and water, respectively, to yield *n*-Hex (4.74 g), MC (5.85 g), EA (18.14 g), *n*-BuOH (42.96 g), and water (167.45 g) fractions. Afterwards, the solvents were evaporated by a vacuum evaporator and freeze drier.

Medium pressure liquid chromatography (MPLC, Grace, Columbia, MD) was employed to fractionate the potential active EA fraction of the SOL extract (1 g). The Grace C18 column (Grace) was used for preparative separation. Methanol and distilled water were used as mobile phase solvents A and B, respectively. Gradient systems were used as follows: 0–15 min, 20% A; 15–100 min, 15–100% A. Flow rate was set at 15 mL/min and the eluent was monitored at 254 and 360 nm. The effluents were collected into test tubes (size is 15 mL) using a fraction collector, and the fractions were manually combined according to the UV detection. Finally, eight fractions were obtained. Compound **2** (81.9 mg) was directly obtained from fraction 5. Fraction 3 was subsequently separated by a Sephadex LH-20 (GE Healthcare Bio-Sciences AB, SE-751 84, Uppsala, Sweden) column eluted with 70% methanol to obtain five subfractions. Subfraction 4 was further purified by high-performance liquid chromatography (HPLC, Gilson, Middleton, WI) coupled with a YMC-Actus Triart C18 column (20 × 250 mm, 5 μm, YMC, Kyoto, Japan) eluted with a solvent mixture of methanol and distilled water (1:1, v/v) to obtain compound **1** (19.91 mg).

### HPLC analysis

HPLC analysis was performed using the Gilson series instrument (Gilson) consisting of a vacuum degasser (BG14), a quaternary pump (321PUMP), an auto-sampler (GX-271), and a variable wavelength detector (UV/VIS-155) system. Separation was carried out on an Eclipse Plus C18 (4.6 × 100 mm, 3.5 μm, Agilent, Santa Clara, CA). The mobile phase consisted of A (0.1% aqueous trifluoroacetic acid) and B (methanol), which was programed as follows: 0–35 min, 20–100% B; 35–45 min, 100% B; 45–50 min, 100–20% B; 50–60 min, 20% B at flow rate of 0.5 mL/min. The UV was set at 254 and 360 nm. The sample injection volume was 20 μL at a column temperature of 30 °C.

### NMR, MS, and FT-IR analysis

The structures of isolated compounds were identified by ^1^H, ^13 ^C nuclear magnetic resonance (NMR), and FT-IR. ^1^H- and ^13 ^C-NMR spectra were recorded at 600 MHz in DMSO-*d*_6_, using a Bruker Avance 600 spectrometer (Bruker Bio, Rheinstetten, Germany), with tetramethylsilane as the internal standard. Signal processing and interpretation were performed using the Bruker DPX 600 MHz package. Moreover, the isolated compounds were analyzed by electron ionization mass spectrometry (EI-MS) performed in a LR-mass equipped with JEOL JMS-700. The mass spectrometer was operated in the negative-ion mode with an electron impact of 70 eV with a direct insertion probe. The ion source was set at 250 °C and the mass range was 50–600 *m/z*. FT-IR spectra were obtained on a Perkin-Elmer 1600 apparatus (Seoul, Korea). Attenuated total reflectance was used for the FT-IR measurements in the frequency range of 4000–400 cm^−1^.

### 3T3-L1 cell culture and adipocyte differentiation

3T3-L1 preadipocytes were cultured, maintained, and differentiated as described by Lee et al. (2011). Briefly, 3T3-L1 cells were grown to confluency at 37 °C under a humidified 5% CO_2_ atmosphere in DMEM, containing 3.7 g/L sodium bicarbonate, 1% P/S, and 10% BCS. Adipocyte differentiation was induced by treating post-confluent cells (2 days after becoming confluent; ‘day 0’) with 10% FBS and a hormonal mixture (MDI), consisting of 0.5 mM IBMX, 1.0 μM DEX, and 1.67 μM insulin. At 2 days after the initiation of differentiation (day 2), the culture medium was replaced with DMEM supplemented with only 1.67 μM insulin and 10% FBS. The cells were cultured medium was replenished every 2 days. Full differentiation was achieved by 8 days. The samples were added to the 3T3-L1 cultures at different concentrations on day 4 after the induction of differentiation.

### Oil Red O staining

The extent of differentiation, as reflected by the amount of lipid accumulation, was determined by Oil Red O staining. Briefly, cells were fixed in 10% formaldehyde in PBS for 1 h, washed with distilled water, and completely dried. Cells were stained with 0.5% Oil Red O solution in 60:40 (v/v) isopropanol:water for 30 min at room temperature and washed four times with water and dried. Differentiation was also monitored under a microscope and quantified by elution with isopropanol and an optical density (OD) measurement at 490 nm.

### MTT assay

The cell viability was investigated using a 3-(4,5-dimethylthiazol-2-yl)-2,5-diphenyltetrazolium bromide (MTT) assay kit (Sigma) according to the manufacturer’s instructions. 3T3-L1 cells (5 × 10^3^/well) were cultured in 96-well plates and treated with samples. The optical density at 490 nm was measured three times using the Multiskan FC Microplate Photometer (Thermo Fisher Scientific, Waltham, MA).

### Statistical analysis

Data from individual experiments are expressed as means ± standard deviation (SD). The results were statistically analyzed by one-way ANOVA and Duncan’s multiple range tests. Statistical significance was accepted at a level of *p* < 0.05.

## Results and discussion

### Anti-adipogenic activity of SOL fractions

**Figure 1.**
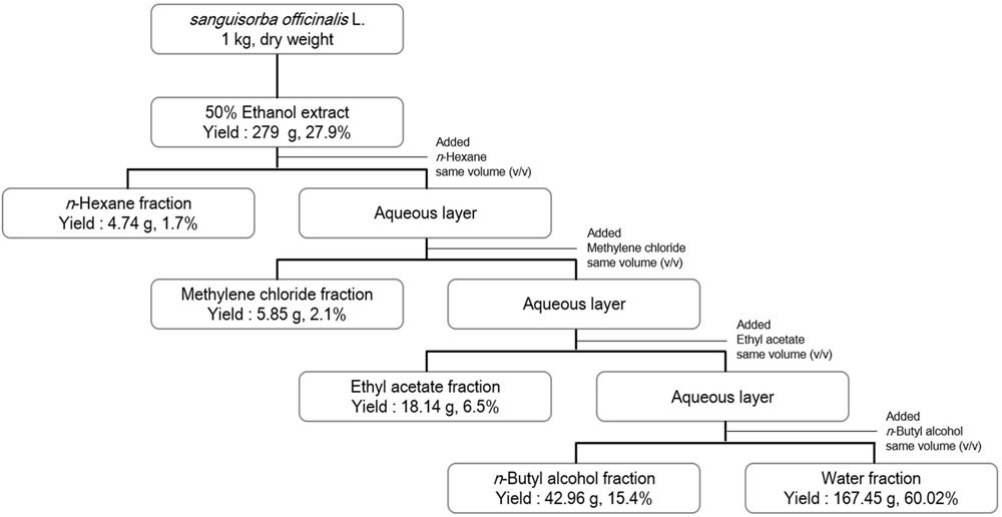
Extraction and fractionation procedures of *Sanguisorba officinalis*.

**Figure 2.**
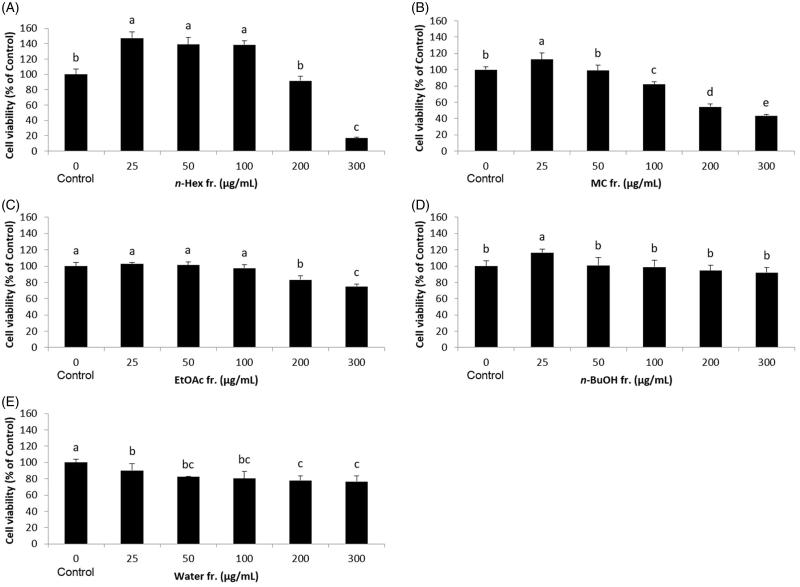
Effect of solvent fractions of *Sanguisorba officinalis* extracts on cell viability of 3T3-L1 preadipocytes. The 3T3-L1 preadipocytes were treated with *Sanguisorba officinalis* 50% ethanol extract (A) *n*-hexane fraction, (B) methylene chloride fraction, (C) ethyl acetate fraction, (D) *n*-butyl alcohol fraction, and (E) water fraction at various concentrations (0, 25, 50, 100, 200, and 300 μg/mL) for 24 h, and the cell viability was determined by MTT assay. Each value is expressed as the mean ± S.D. Values with different superscripts are significantly different at *p* < 0.05.

**Figure 3.**
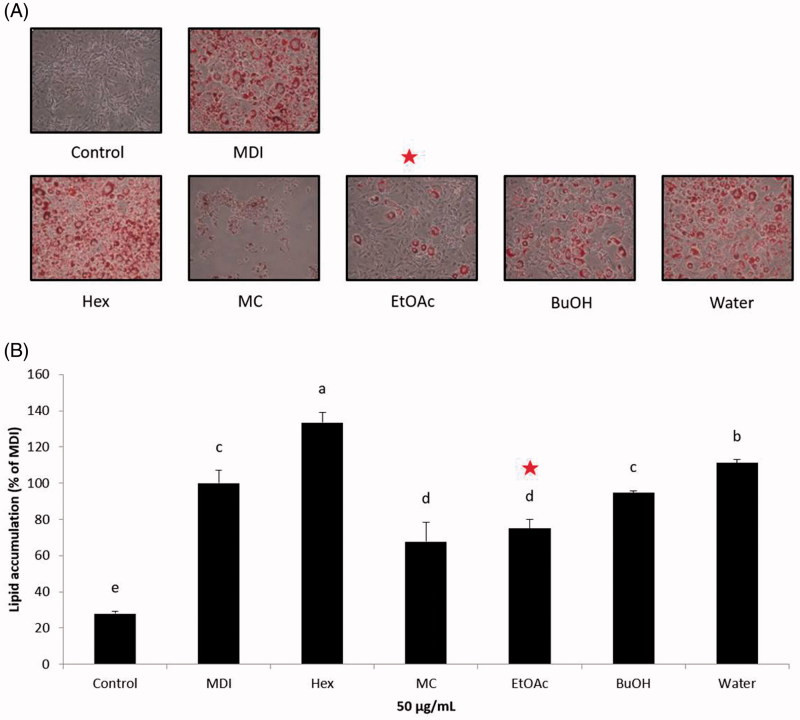
Effect of solvent fractions of *Sanguisorba officinalis* extracts on lipid accumulation in 3T3-L1 adipocytes. (A) The morphological changes associated with cell differentiation were photographed after Oil Red O staining. (B) Stained lipids were extracted and quantified by measuring absorbance at 570 nm. Each value is expressed as the mean ± S.D. Values with different superscripts are significantly different at *p* < 0.05. Control: undifferentiated preadipocyte; MDI: differentiated adipocyte; Hex: adipocyte was treated with *n*-hexane fraction; MC: adipocyte was treated with methylene chloride fraction; EtOAc: adipocyte was treated with ethyl acetate fraction, BuOH: adipocyte was treated with *n*-butyl alcohol fraction; and Water: adipocyte was treated with water fraction. The red star indicates the target fraction.

In modern society, obesity seriously threatens public health. To develop anti-adipogenesis agents for preventing obesity, 3T3-L1 cells serve as a well-documented model system. As shown in Figure 1, five fractions, namely *n*-Hex, MC, EA, *n*-BuOH, and water fractions, were prepared and their anti-adipogenic effects were evaluated by Oil Red O staining during the differentiation of 3T3-L1 preadipocytes. The cell viability of 3T3-L1 cells was first investigated using the MTT assay. As shown in Figure 2, the cytotoxic effects of *n*-Hex, EA, MC, and water fractions on 3T3-L1 cells were observed at the concentrations of 300, 200, 100, and 25 μg/mL, respectively, whereas the *n*-BuOH fraction does not show any cytotoxic effects on 3T3-L1 cells. Therefore, a concentration of 50 μg/mL was used for subsequent experiments. When the preadipocytes were differentiated into adipocytes, morphological alterations were observed due to the accumulation of lipid droplets in the cytoplasm. Figure 3 shows the effects of SOL fractions on the adipogenesis of 3T3-L1 cells. The MC and EA fractions significantly suppressed the lipid accumulation in differentiated 3T3-L1 cells compared to the MDI control. Considering that the anti-adipogenic effect of the EA fraction showed no significant differences compared with the MC fraction, and the cytotoxic effect of the EA fraction was lower than that of the MC fraction, the EA fraction was selected for further bioassay-guided isolation.

### Isolation and identification of isorhamnetin-3-O-d-glucuronide (1) and ellagic acid (2)

The anti-adipogenic activity of the EA fraction may be due to bioactive compounds present within it. This result exhibited consistency with a previous report (Jung et al. 2016), as it suggested that a 50% EtOH extract of SOL inhibited the adipogenesis associated with reduced expression of adipogenic transcription factors such as PPAR-γ and C/EBPα proteins in 3T3-L1 cells. Rayalam et al. (2008) showed that natural compounds inhibited adipogenesis due to their bioactive compounds, including phenolic compounds and flavonoids. The anti-adipogenic activity of SOL fractions may be influenced by major phenolic compounds within these fractions.

**Figure 4.**
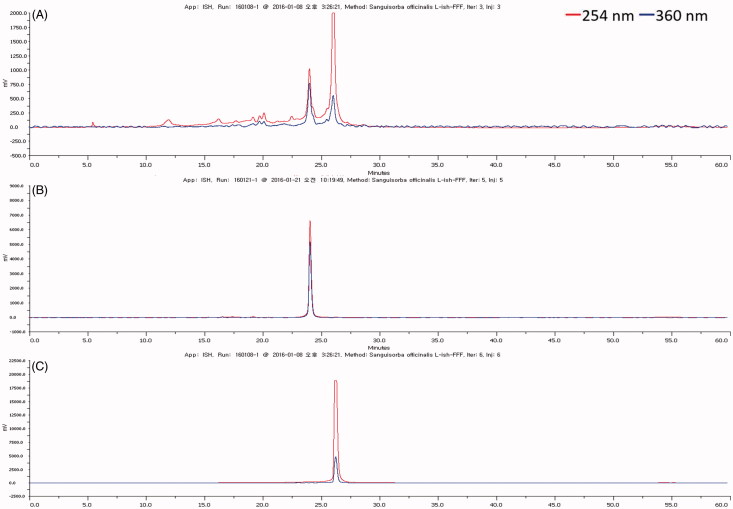
HPLC chromatograms of compounds **1** and **2** of ethyl acetate fraction. (A) Ethyl acetate fraction of *Sanguisorba officinalis* 50% ethanol extract, (B) Compound **1**, and (C) Compound **2**.

As shown in Figure 4(A), the HPLC pattern of EA fraction of SOL is very simple, in which only two major peaks are present, indicating that these two compounds might be responsible for its anti-adipogenic effect. Thus, as shown in Figure 4(B,C), these two compounds were isolated and purified using chromatography as described in section: Extraction, fractionation, and isolation of bioactive compounds from SOL. The two isolated compounds were identified to be as isorhamnetin-3-*O*-d-glucuronide **(1)** and ellagic acid **(2)** using multiple spectral techniques.

**Figure 5.**
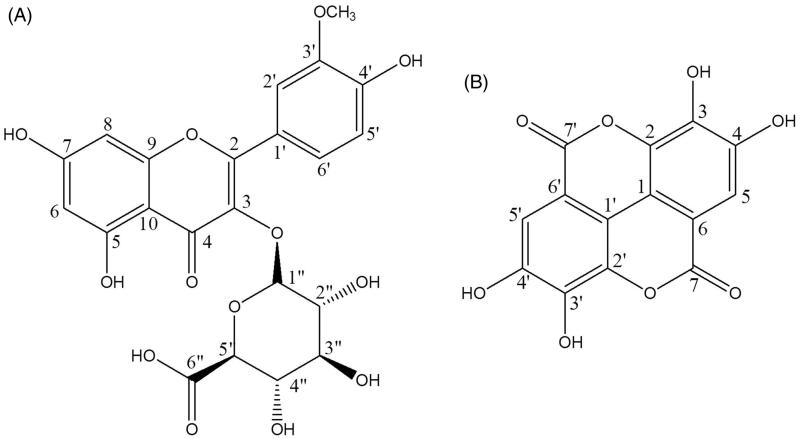
Structures of compound **1** (isorhamnetin-3-*O*-d-glucuronide) and compound **2** (ellagic acid).

Compound **1** was established to possess a molecular weight of 492.39 and a chemical formula of C_22_H_20_O_13_ on the basis of its IR spectrum, ^1^H-NMR, ^13 ^C-NMR, and MS. The IR spectrum displayed absorption bands for 3406 (–OH), 2924 (C–H), 1652 (C = O) 1604 (aromatic C = C), 1499 (aromatic C = C), 1443 (aromatic C = C), 1358 (–C–OH), 1301 (–C–OH), 1272 (–C–OH), 1202 (–C–OH), 1171 (–C–OH), 1114 (–C–OH), 1086 (–C–OH). ^1^H-NMR (600 MHz, DMSO-*d*_6_) was 8.23 (dd, 1H, *J* = 8.47, 2.10 Hz, H-6′), 7.36 (d, 1H, *J* = 7.26 Hz, H-2′), 6.83 (d, 1H, *J* = 8.47 Hz, H-5′), 6.38 (d, 1H, *J* = 1.26 Hz, H-8), 6.19 (d, 1H, *J* = 1.27 Hz, H-6), 5.25 (d, 1H, *J* = 7.51 Hz, H-1″), 3.23–3.41 (m, 6H), 3.17 (s, 3H, –OCH_3_), respectively. ^13 ^C-NMR (150 MHz, DMSO-*d*_6_) was *δ* 177.90 (C-4), 173.17 (C-6″), 166.00 (C-7), 161.44 (C-5), 157.88 (C-2), 157.04 (C-9), 149.00 (C-3′), 145.34 (C-4′), 134.47 (C-3), 121.20 (C-2′), 120.96 (C-1′), 118.29 (C-6′), 115.87 (C-5′), 104.04 (C-10), 103.35 (C-1″), 99.58 (C-6), 94.30 (C-8), 77.08 (C-3″), 74.81 (C-5″), 74.57 (C-2″), 72.27 (C-4″), 49.07 (–OCH_3_), respectively. Compound **1** can be identified by heteronuclear single-quantum correlation (HSQC) and heteronuclear multiple-bond correlation (HMBC) spectra. The HMBC spectrum was (1) H-6 proton correlated with C-5, C-7, C-8, and C-10 (2) H-8 proton correlated with C-6, C-7, C-9, and C-10 (3) H-2′ proton correlated with C-2, C-3′, and C-6′ (4) H-5′ proton correlated with C-1′, C-3′, and C-4′. Consequently, compound **1** was confirmed as isorhamnetin-3-*O*-d-glucuronide [6-((5,7-dihydroxy-2-(4-hydroxy-3-methoxyphenyl)-4-oxo-4H-chromen-3-yl)oxy)-3,4,5-trihydroxytetrahydro-2H-pyran-2-carboxylic acid] (Figure 5(A)).

Compound **2** was established as possessing a molecular weight of 302.12 and a chemical formula of C_14_H_6_O_8_. Its IR spectrum displayed absorption bands for 3300–2700 (–OH), 3021 (C–H), 2926 (C–H), 1713 (C = O), 1616 (aromatic C = C), 1580 (aromatic C = C), 1506 (aromatic C = C), 1440 (aromatic C = C), 1409 (aromatic C = C), 1391 (aromatic C = C), 1310 (–C–OH), 1290 (–C–OH), 1185 (–C–OH), 1092 (–C–OH). ^1^H-NMR (600 MHz, DMSO-*d*_6_) was *δ* 7.46 (s, 2H, ArH, H-5, 5′), 10.68 (s, 4H, –OH), respectively. ^13 ^C-NMR (150 MHz, DMSO-*d*_6_) was *δ* 159.60 (C-7, 7′), 148.60 (C-4, 4′), 140.11 (C-3, 3′), 136.85 (C-2, 2′), 112.80 (C-1, 1′), 110.68 (C-5, 5′), 108.06 (C-6, 6′). Consequently, compound **2** was confirmed as ellagic acid [2,3,7,8-tetrahydroxychromeno(5,4,3-cde)chromene-5,10-dione] (Figure 5(B)).

### Anti-adipogenic activity of isorhamnetin-3-O-d-glucuronide (1) and ellagic acid (2)

**Figure 6.**
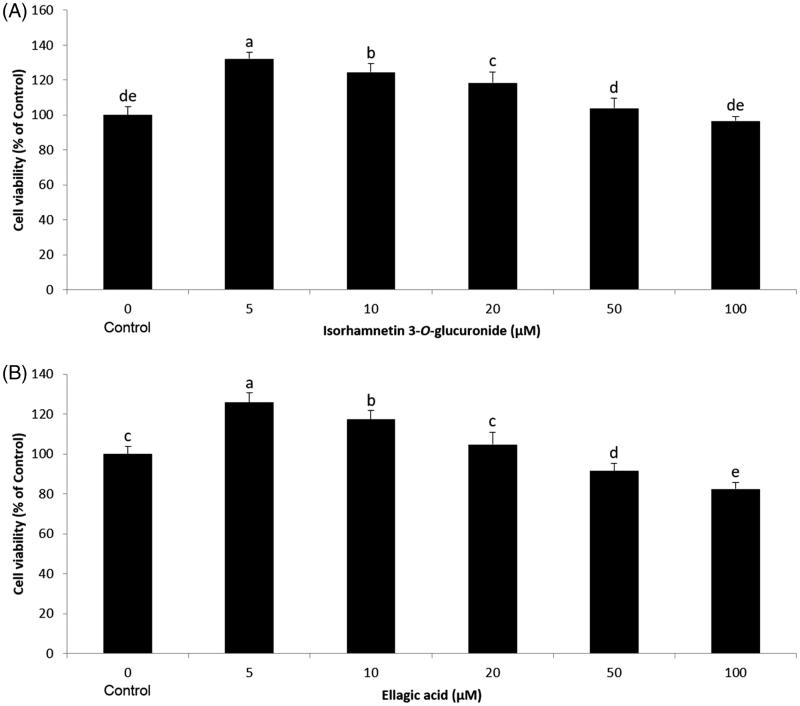
Effect of compounds on cell viability of 3T3-L1 preadipocytes. The 3T3-L1 preadipocytes were treated with ethyl acetate fraction (A) isorhamnetin-3-*O*-d-glucuronide and (B) ellagic acid at various concentrations (0, 5, 10, 20, 50, and 100 μM) for 24 h, and the cell viability was determined by MTT assay. Each value is expressed as the mean ± S.D. Values with different superscripts are significantly different at *p* < 0.05.

**Figure 7.**
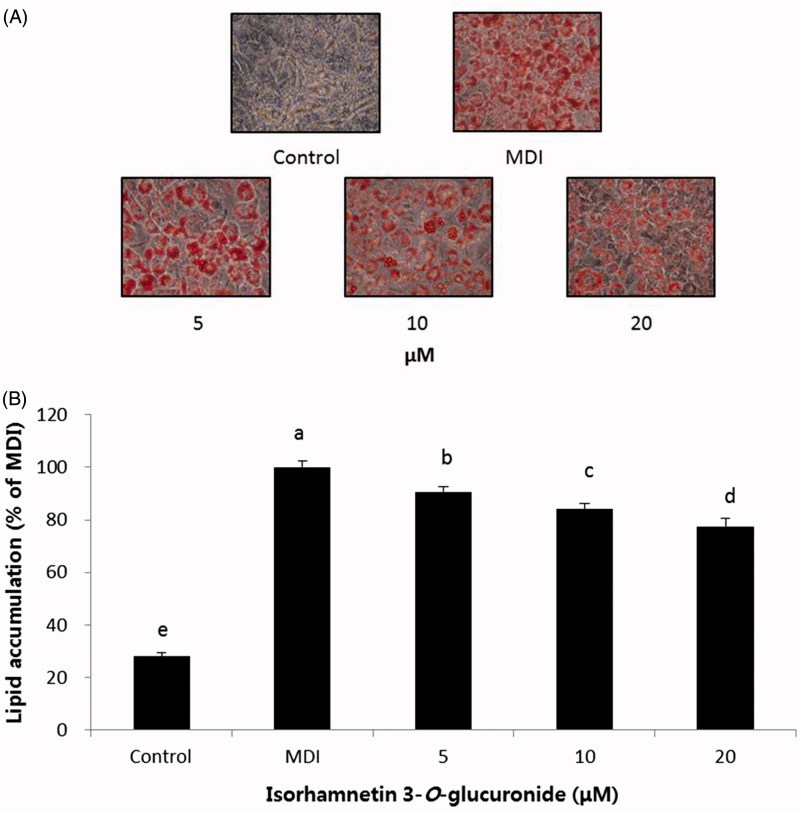
Effect of compound **1** on lipid accumulation in 3T3-L1 adipocytes. (A) The morphological changes associated with cell differentiation were photographed after Oil Red O staining. (B) Stained lipids were extracted and quantified by measuring absorbance at 570 nm. Each value is expressed as the mean ± S.D. Values with different superscripts are significantly different at *p* < 0.05. Control: undifferentiated preadipocyte; MDI: differentiated adipocyte.

**Figure 8.**
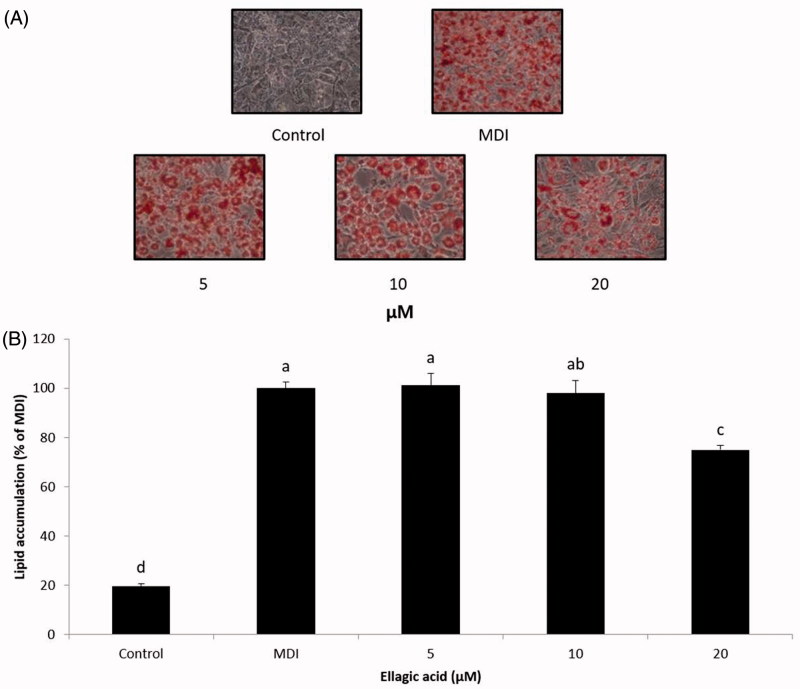
Effect of compound **2** on lipid accumulation in 3T3-L1 adipocytes. (A) The morphological changes associated with cell differentiation were photographed after Oil Red O staining. (B) Stained lipids were extracted and quantified by measuring absorbance at 570 nm. Each value is expressed as the mean ± S.D. Values with different superscripts are significantly different at *p* < 0.05. Control: undifferentiated preadipocyte; MDI: differentiated adipocyte.

We further examined the effect of isorhamnetin-3-*O*-d-glucuronide and ellagic acid on adipogenesis. Preadipocytes were differentiated with MDI in the presence of various concentrations (5, 10, 20 μM) of isorhamnetin-3-*O*-d-glucuronide and ellagic acid, and cellular lipids were stained using the Oil Red O staining method on day 8. The isorhamnetin-3-*O*-d-glucuronide and ellagic acid were not toxic to the 3T3-L1 cells within the experimental range of concentrations (Figure 6). No change in cell morphology was observed in the microscopic analysis (data not shown). Concentrations of 5, 10, and 20 μM were chosen for subsequent experiments. Our results showed that both isorhamnetin-3-*O*-d-glucuronide and ellagic acid at a concentration of 20 μM significantly inhibited lipid accumulation compared to the MDI control (Figures 7 and 8). These results are in good agreement with a previous study by Woo et al. (2015), in which ellagic acid inhibits adipocyte differentiation by suppressing early adipogenic events and cell cycle arrest. Furthermore, Lee et al. (2009) reported that isorhamnetin inhibits adipogenesis through the down-regulation of PPAR-γ and C/EBPα. The anti-adipogenic activity of ellagic acid was higher than that of isorhamnetin-3-*O*-d-glucuronide. Our observations suggest that the anti-adipogenic activity of SOL extract and fractions may due to their bioactive compounds, including isorhamnetin-3-*O*-d-glucuronide and ellagic acid. Therefore, SOL could be an excellent natural anti-adipogenic ingredient. Our results provide useful data in the development of a potent anti-obesity agent.

## Conclusions

The bioassay-guided fractionation, isolation, and identification of anti-adipogenic bioactive compounds in *Sanguisorba officinalis* were investigated by using effective differentiation of 3T3-L1 cells to adipocytes. Isorhamnetin-3-*O*-d-glucuronide **(1)** and ellagic acid **(2)** were isolated and identified as major anti-adipogenic compounds.
